# SR5AL serves as a key regulatory gene in lycopene biosynthesis by *Blakeslea trispora*

**DOI:** 10.1186/s12934-022-01853-x

**Published:** 2022-06-25

**Authors:** Qiang Wang, Yulong Chen, Qingxiang Yang, Jihong Zhao, Lingran Feng, Min Wang

**Affiliations:** 1 College of Life Sciences, Henan Normal University, Xinxiang, 453007 China; 2 Henan International Joint Laboratory of Agricultural Microbial Ecology and Technology (Henan Provincial Department of Science and Technology), Henan Normal University, Xinxiang, 453007 China

**Keywords:** Trisporic acids, Lycopene biosynthesis, Steroid 5α-reductase, *Sex*M gene, *Blakeslea trispora*

## Abstract

**Background:**

Trisporic acids are considered to be key regulators of carotenoid biosynthesis and sexual reproduction in zygomycetes, but the mechanisms underlying this regulation have not been fully elucidated.

**Results:**

In this study, the relationships between trisporic acids and lycopene synthesis were investigated in *Blakeslea trispora*. The lycopene concentration in single fermentation by the (−) strain with the addition of 24 μg/L trisporic acids was slightly higher than that observed in mated fermentation. After transcriptomic analysis, a steroid 5α-reductase-like gene, known as SR5AL in *B. trispora,* was first reported. 5α-Reductase inhibitors reduced lycopene biosynthesis and downregulated the expression of sex determination and carotenoid biosynthesis genes. Overexpression of the SR5AL gene upregulated these genes, regardless of whether trisporic acids were added.

**Conclusion:**

These findings indicated that the SR5AL gene is a key gene associated with the response to trisporic acids.

**Supplementary Information:**

The online version contains supplementary material available at 10.1186/s12934-022-01853-x.

## Background

Lycopene naturally exists in some plants and microorganisms and gives them red color, such as tomatoes, *Blakeslea trispora*, *Rhodotorula mucilaginosa*, and *Dunaliella salina* [1, 2]. It is a good coloring agent and antioxidant, and is widely used in pharmaceutical, food, feed and other fields. *B. trispora* is the main fungus for industrial production of carotenoids and β-carotene is its main final product under common conditions, while lycopene is an intermediate product. To produce lycopene, cyclase inhibitor or genetic modification is required to prevent lycopene from forming β-carotene [3]. *B. trispora* is a zygomycotal fungus with two mating types, termed ‘(+)’ and ‘(−)’. The (−) mating type is the main producer of carotenoids, but the (+) mating type is essential partner for the overproduction of carotenoids [4]. The lack of trisporic acids is considered to be the key reason why carotenoids cannot be largely synthesized in single culture with *B. trispora* (−) [5, 6]. Trisporic acids are downstream products of β-carotene, which also depends on the joint cultivation of heterothallic (+) and (−) strains. This is because the mating type (−) is unable to convert its own precursor into trisporic acids. The precursor trisporol B produced by the mating type (−) needs to be taken up by the (+) strain and subsequently converted to trisporic acids [7].

Trisporic acids, serving as sex hormones, induce the first steps of sexual differentiation and maintain the development of sexual structures in the members of the order Mucorales, namely, *B. trispora*, *Phycomyces blakesleeanus*, and *Mucor mucedo* [8–10]. Trisporic acids could induce sexual cytodifferentiation of mucoraceous fungi, such as development of sexual structures, fusion of gametangia, and formation of zygotes. Sexual reproduction is governed by a sex locus in Mucorales (*sex*P gene in the (+) strain and *sex*M gene in the (−) strain). Similar to Mucorales fungi, the sex loci in *B. trispora* contains a high-mobility group transcription factor which is flanked by a putative triose phosphate transporter gene and an RNA helicase gene [11]. Notably, the expression patterns of the *sex*P and *sex*M genes are very different. The *sex*M gene is expressed during mated culture or the addition of exogenous trisporic acids, while the *sex*P gene is expressed during both single and mated culture [12, 13]. Exposure to trisporic acids was observed to considerably increase the expression of the *sex*M gene, but the *sex*P gene showed no significant change [12]. In addition, the *sex*M protein but not the *sex*P protein harbors a nuclear localization sequence and is transported to the nuclei [12]. These reports suggest that the two sex genes may have different functions in sexual reproduction. The *sex*M gene appears to be more closely related to trisporic acids than the *sex*P gene. However, the direct connection between the sex determinant gene and trisporic acids has not been fully elucidated to date.

Accumulating evidence shows that trisporic acids are the key regulators of lycopene and other carotenoids [6, 8, 14]. Carotenoids are the precursors of trisporic acids, and trisporic acids, in turn, are the key regulating factors for carotenoid synthesis. The carotenoid concentration fermented by a single culture of *B. trispora* (−) in the presence of trisporic acids is almost equivalent to that of mixed fermentation [15]. The contributions of the mating type (+) to lycopene biosynthesis in mated fermentation can largely be replaced by trisporic acids [6, 15]. The steady-state transcription levels of the lycopene and β-carotene synthesis genes *car*B and *car*RA were observed to increase in the presence of trisporic acids [16]. In addition, the transcription of the *tsp3* gene encoding β-carotene oxygenase was also induced by trisporic acids in *B. trispora* [17]. Metabolic level changes of *B. trispora* in response to trisporic acids showed that trisporic acids are global regulators, and their regulation involves multiple metabolic pathways, such as the metabolism of fatty acids, carbohydrates, and amino acids [14].

To date, it has only been suggested that trisporic acids can increase the expression of the *sex*M gene and carotenoid synthesis genes and the production of carotenoids. However, the regulatory mechanisms underlying the effects of trisporic acids on these genes have not been fully elucidated. In addition, the product of *sex*M gene is a transcription factor and located in the nucleus. The regulatory effect of the *sex*M protein on the expression of the genes involved in the biosynthesis of carotenoids and trisporic acids has not been fully elucidated. The regulation mode and key genes for controlling carotenoid biosynthesis in mated culture or responding to trisporic acids have not been determined to date. In this study, the changes at the transcriptional level in *B. trispora* (−) with and without trisporic acids were characterized by RNA-seq and validated by reverse transcription qPCR. Next, one of the candidate genes, the steroid 5α-reductase-like gene (SR5AL), was further analyzed to study its function in lycopene biosynthesis. Finally, a hypothesis regarding how trisporic acids regulate lycopene synthesis was proposed. The aims of this study were to identify some key regulatory genes in response to trisporic acids and to suggest a mechanism by which trisporic acids regulate the synthesis of lycopene.

## Methods

### Microorganisms and culture conditions

*Blakeslea. trispora* NRRL 2895 (+) mating type and the N6 (−) mating type were used in this study. The medium compositions and culture conditions are the same as our previous paper [18]. Briefly, the spores of the (+) and (−) strains were collected on their respective solid medium; then, the spores were inoculated in liquid seed medium and cultured separately; finally, they were inoculated into the fermentation medium for mixed fermentation. To obtain lycopene instead of β-carotene, the cyclase inhibitor 2-methylimidazole at a concentration of 0.3 g/L was added after 48 h of fermentation.

### Extraction of trisporic acids

The procedures for the extraction of trisporic acids were according to Schachtschabel et al. and Schimek et al. [8, 19]. Briefly, the filtrate separated from the mated culture of *B. trispora* was adjusted to pH 8.0 and subsequently extracted with organic phase (trichloromethane: 2-propanol = 20:1, v/v). The water phase was separated and then adjusted to pH 2.0 and later extracted once again with the same organic phase. Finally, the extracts in organic phase with trisporic acids were dried in a rotary vacuum concentrator and then redissolved in 75% (v/v) ethanol. Trisporic acid was quantified by measuring the absorbance at 325 nm in ethanol. Concentration of trisporic acid were calculated using specific extinction coefficients (${E}_{325 nm}^{1\%, 1 cm}=572$) [14, 20].

### Transcriptome sequencing and data analysis

#### cDNA library preparation and sequencing

Total RNA of *B. trispora* (−) with and without trisporic acids treatment were extracted using a Spin Column Fungal Total RNA Purification Kit (Sangon, Shanghai, China), according to the manufacturer’s procedures. After standard processing procedures, cDNA library were prepared, and the average insert size for the paired-end libraries was 300 bp (± 50 bp). Then, paired-end sequencing was performed on the Illumina HiSeq 4000 platform. Raw RNA-seq datasets of *B. trispora* are deposited in the sequence read archive (SRA) database (Accession Number PRJNA728833).

#### Sequence assembly and unigene annotation

Raw reads were filtered to remove adaptors, reads with more than 5% unknown nucleotides, and other low-quality reads. After QC filtering, de novo assembly of the transcriptome was performed with Trinity 2.4.0. All assembled unigenes were aligned against the nonredundant protein, Gene Ontology, SwissProt, Kyoto Encyclopedia of Genes and Genomes and eggNOG databases using DIAMOND with a threshold E-value < 0.00001.

#### Identification and analysis of differentially expressed genes

The expression levels of the unigenes were calculated by using the TPM method. Fold changes (FCs) were calculated by comparing the TPM values of unigenes. The *p*-value and false discovery rate (FDR) were applied to determine the differential expression of unigenes. Unigenes with |log_2_FC|≥ 1, *p* < 0.05, and FDR ≤ 0.001 were designated differentially expressed genes (DEGs). Then, the DEGs were subjected to GO and KEGG pathway enrichment analysis by using DAVID [21]. FDR < 0.05 was set as the cut-off criterion for the two analyses.

#### SR5AL gene cloning

According to the sequence of Unigene4532, a BLAST procedure was performed to find the homologous DNA region in the genomes of *B. trispora* F921, F986, and NRRL 2456 (https://genome.jgi.doe.gov/portal/). Then, the primers SR5AL-F (5′-CAGGATGATGAAGACTCAAGGATAG-3′) and SR5AL-R (5′-AGAAAGGAAAGGATGAGAACCG-3′) were designed to amplify the SR5AL gene. PCR products were purified by agarose gel electrophoresis and subsequently sequenced (Sangon, China).

#### Construction of SR5AL-gene overexpression strain

##### Construction of overexpression vector

The plasmid pBARGPE1-Hygro, a filamentous fungus-*E. coli* shuttle expression vector, was used as the vector backbone (obtained from Miaoling Biotechnology Company, Wuhan, China). It replicates in *E. coli* and carries ampicillin resistance. In filamentous fungi, gpdA promoter can initiate the expression of foreign genes and positive clones can be selected with hygromycin. Total RNA of *B. trispora* (−) was extracted by using a Fungal RNA Kit (Omega Bio-Tek, USA) and subsequently reverse-transcribed to cDNA with a FastKing RT Kit (Tiangen, China). The DNA fragment containing the cDNA of the SR5AL gene was amplified by PCR using primers SR5ALC-F (5′-CCGGAATTCCAATTATTGCTTTTCTGCTCG-3′) and SR5ALC-R (5′-GGGGTACCGTATGTTGAAAGACAGGGCTC-3′). The fragment was double-digested with BamHI and EcoRV and inserted into the plasmid pBARGPE1-Hygro. The recombinant plasmid was named pBARGPE1-Hygro-SR5AL (As shown in Additional file 1: Fig. S1). Expression of the SR5AL gene was under the control of the gpdA promoter and trpC terminator. The constructed plasmid was transformed into *Escherichia coli* DH5α and confirmed by sequencing.

##### Preparation of protoplasts, transformation and regeneration

The *B. trispora* (−) protoplasts were prepared as described by Wang Yanlong et al. [22]. Mycelia were collected after 24 h of culture and subsequently treated with enzymatic solution (1.5% lysozyme, 3% lyticase, 1.5% snail enzyme, and 1% cellulose dissolved in 0.6 mol/L sucrose). After 2 h of incubation, the mixture was filtered through 200 and 500 mesh screens and centrifuged. The precipitate was resuspended and diluted to 10^7^ cells/mL by using 0.6 mol/L sucrose. The pBARGPE1-Hygro-SR5AL and pBARGPE1-Hygro vectors (10 μg) were mixed with 200 μL protoplasts, and 50 μL polyethylene glycol was added. The mixture was placed in an ice bath for 30 min and subsequently held at room temperature for 20 min after adding 1 mL polyethylene glycol again. The protoplasts were then transferred into 1 mL regeneration medium for 2 h (25 °C, 100 r/min). Protoplasts (100 μL) were spread on regeneration medium supplemented with hygromycin to select transformants. The transformants carrying pBARGPE1-Hygro-SR5AL and pBARGPE1-Hygro were named btpHY and btpH, respectively.

##### Reverse transcription Quantitative PCR (RT-qPCR)

RT-qPCR experiments were performed to determine the expression of the *hmg*R, *car*RA, *car*B, SR5AL and *sex*M genes. Total RNA of *B. trispora* (−) was extracted by using a Fungal RNA Kit (Omega Bio-Tek, USA) and subsequently reverse-transcribed to cDNA with a FastKing RT Kit (Tiangen, China). Real-time PCRs were conducted with AceQ qPCR SYBR Green Master Mix (Vazyme Biotech, Nanjing, China) in a LightCycler instrument (Roche). The PCR reaction contained 10 µL 2 × SYBR Green Mix, 1 µL cDNA, 0.4 µL forward primer and 0.4 µL reverse primer, and nuclease-free water in a final volume of 20 µL. The primers used for qPCR are listed in Table 1. All results obtained by real-time PCR were normalized to the *tef*1 gene and compared with the corresponding control (value = 1) and the relative fold changes were calculated using the 2^−ΔΔCt^ method.

**Table 1:** Oligonucleotide primers used for qPCR

Primer	Sequence (5′ → 3′)	Amplicon length (bp)
*hmg*R-for	AAACGATGGATTGAACAAGAGGG	113
*hmg*R-rev	TAGACTAGACGACCGGCAAGAGC	
*car*RA-for	CCGTTTCACTCACAGCAAGA	135
*car*RA-rev	GACAGCCACAACACAAGTAGGA	
*car*B-for	AGACCTAGTACCAAGGATTCCACAA	92
*car*B-rev	AGAACGATAGGAACACCAGTACCTG	
SR5AL-for	TCCCTTTTTTTTACATTTCGTTTTGG	180
SR5AL-rev	ATACCTTGGTTGTTTTGAGAGCCCT	
*sex*M-for	AACTCTCTGCTCTCATTGGTG	136
*sex*M-rev	GCTTTGTTTTTTTCTTCGCT	
*tef*1-for	AACTCGGTAAGGGTTCCTTCAAG	138
*tef*1-rev	CGGGAGCATCAATAACGGTAAC	

##### Extraction and analysis of lycopene

The extraction and concentration determination of lycopene are the same as our previous paper [18].

##### Statistical analysis

Statistical analyses were performed using R (v4.0.1). Comparisons between groups were based on the two-sided Wilcoxon rank-sum test. All *P* values were calculated using two-sided tests, and *P* < 0.05 was considered to be statistically significant.

## Results

### Effects of trisporic acids on lycopene production and the expression of carotenogenic genes

**Fig. 1:**
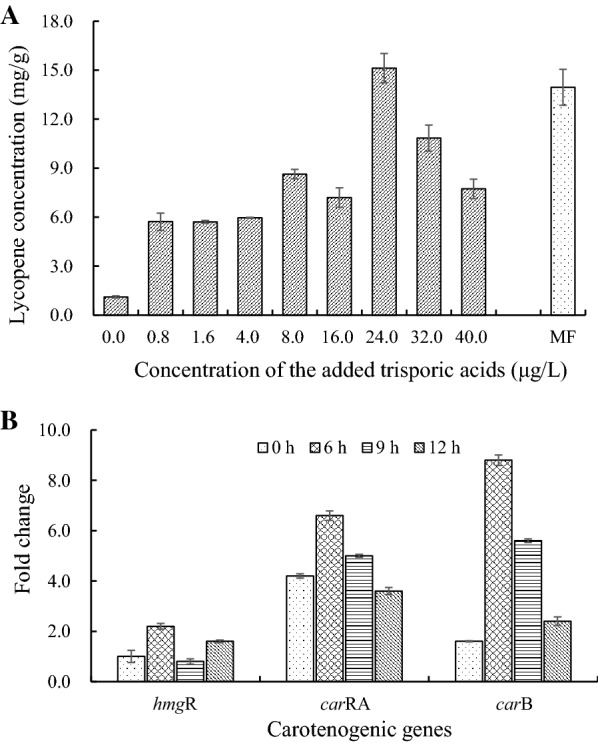
Effects of trisporic acids on lycopene biosynthesis in *B. trispora*. **A**: lycopene concentration; **B**: carotenogenic gene expression with 24 μg/L trisporic acids. MF: mated fermentation. Single fermentation by the (−) strain in all tests except MF. *hmg*R gene transcripts at 0 h was used as the calibrator (value = 1) for relative quantification

As shown in Fig. 1A, the lycopene yield in the (−) mating type was only 1.12 mg/g dry biomass in the absence of the (+) partner, which was considerably less than the production obtained from mixed fermentation. The addition of trisporic acids has a significant effect on the synthesis of lycopene. The maximum lycopene concentration (15.12 mg/g dry biomass) of the (−) mating type was obtained when 24 μg/L trisporic acids were added, which was even slightly higher than that observed with mated fermentation (13.95 mg/g dry biomass). To determine the extraction time of transcriptome RNA, the expression levels of three carotenogenic genes (*hmg*R, *car*RA, and *car*B) were measured at different times after adding trisporic acids. As shown in Fig. 1B, the expression of carotenogenic genes was clearly increased under stimulation with trisporic acids, especially the *car*RA and *car*B genes. The highest relative abundances for the three genes were observed when trisporic acids were added for 6 h. Therefore, to facilitate transcriptome analysis, the extraction time of total RNA of the (−) mating type was chosen to be 6 h after adding trisporic acids at 24 h of fermentation.

### Transcriptome analysis of B. trispora in response to trisporic acids

To explore the transcriptional changes of *B. trispora* (−) in response to trisporic acids, RNA sequencing of the (−) mating type with and without the addition of trisporic acids was performed. After a series of bioinformatic treatments and analyses (as described in the Methods), a total of 28,399 unigenes were generated, among which 59.32% of the unigenes presented a length > 1000 bp (N_50_ = 2080). The fragments per kilobase per million mapped fragments method (FPKM) was used to calculate the expression values of the genes. A total of 1026 differentially expressed genes (DEGs, with fold changes ≥|2|, p < 0.05, FDR ≤ 0.001) were obtained. Among these DEGs, 487 genes were upregulated, and 539 genes were downregulated, in *B. trispora* (−) in response to trisporic acids compared with the control group. In addition, to validate the RNA-seq results, the expression levels of four DEGs, *car*B, *car*RA, SR5AL (steroid 5α-reductase like gene), and *sex*M (sex determining gene), were quantified by RT-qPCR (Additional file 1: Fig. S2). The results showed that the gene expression patterns of these DEGs were consistent with those obtained by RNA-seq, supporting the reliability and accuracy of the RNA-seq analysis results.

**Fig. 2:**
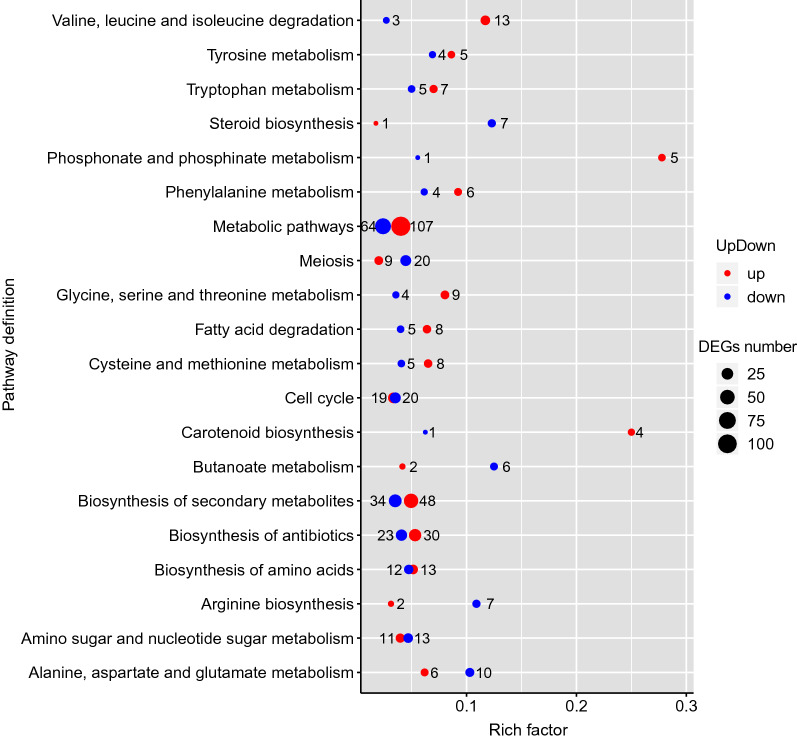
KEGG annotation of DEGs. The rich factor indicates the ratios of up- or downregulated DEGs to all annotated genes

To explore the functions of DEGs, KEGG analysis was performed. The results showed that 290 upregulated genes and 324 downregulated genes were grouped into 110 known metabolic or signaling pathway classes. As shown in Fig. 2, the pathways relevant to valine, leucine and isoleucine degradation, phosphonate and phosphinate metabolism, and carotenoid biosynthesis included more upregulated DEGs in *B. trispora* (−) treated with trisporic acids. Downregulated DEGs prevailed in the pathways associated with steroid biosynthesis, butanoate metabolism, and arginine biosynthesis.

The top 10 of up- and downregulated DEGs were shown in Additional file 1: Fig. S3. Among these upregulated DEGs, Unigene1762_All (encoding carotene oxygenase), CL1473.Contig2_All (encoding carotenoid cleavage dioxygenase 1), and Unigene6321_All (sex determining gene) are related to sexual reproduction. Genes coding for transcription factors (HMG-box and GATA) also showed high fold changes. Notably, although there is no report that Unigene4532_All (encoding steroid 5α-reductase like gene) is related to the synthesis of lycopene, it shows the highest fold changes, and steroid 5α-reductase is related to the biosynthesis of hormones. Among the top 10 downregulated DEGs, some genes are related to protein post-translational modifications, such as CL464.Contig12_All (encoding serine/threonine protein kinase), CL435.Contig20_All (encoding protein phosphatase).

### Roles of steroid 5α-reductase-like gene (SR5AL) in lycopene biosynthesis and sexual reproduction

Based on the fold changes and the metabolic pathways involved, some unigenes were selected as candidate genes, such as Unigene4532_All (SR5AL, steroid 5α-reductase like gene, fold change = 8.79), Unigene1762_All (*tsp*3, putative carotene oxygenase, fold change = 8.71), and Unigene6321_All (*sex*M, sex determining gene, fold change = 7.14). Later, the results showed that SR5AL is likely to be one of the key genes associated with the response to trisporic acid stimulation.

### Characterization of steroid 5α-reductase like gene

**Fig. 3:**
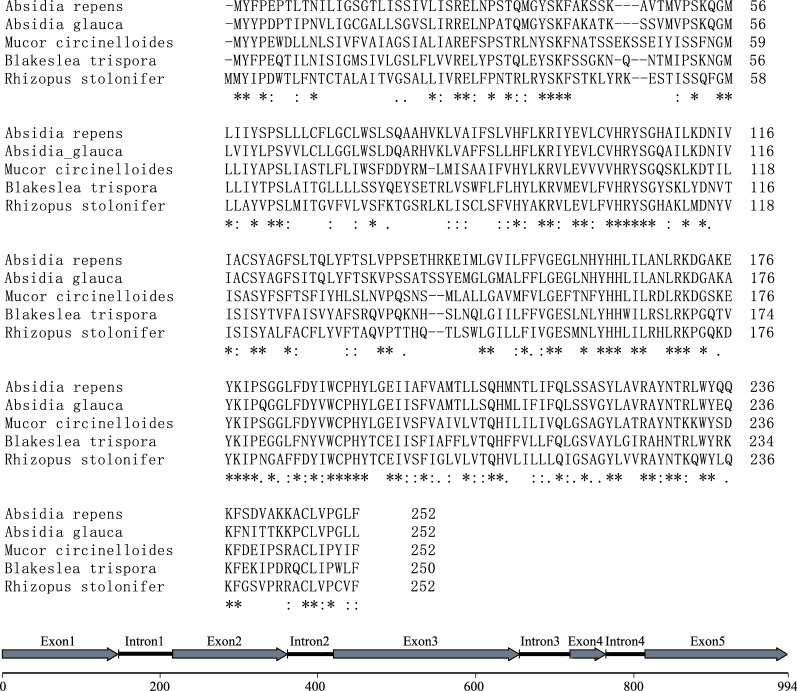
Characterization of the steroid 5α-reductase-like gene

According to the cDNA sequences of Unigene4532, homologous DNA regions were searched from the genomes of *B. trispora* F921, F986, and NRRL 2456 (https://genome.jgi.doe.gov/portal/). Next, a 2154-bp DNA region containing Unigene4532 was amplified by PCR using *B. trispora* N6 (used in this study) genomic DNA as a template. A 994-bp open reading frame (starting with ATG at 799 bp and ending with TGA at 1792 bp) was found, which contained five exons and four introns. Intron splicing was confirmed by cDNA sequence. The translated amino acid sequence is highly similar to the sequence of steroid 5α-reductase from *Absidia repens*, *A. glauca*, *Mucor circinelloides*, and *Rhizopus stolonifer* (Fig. 3). This gene was named the SR5AL gene (steroid 5α-reductase-like gene). The TATA box was detected at the 5′-flanking region (TATATCT at 376 bp), and a polyadenylation signal was found at the 3’-flanking region (AATAAA at 1867 bp).

### Effects of steroid 5α-reductase inhibitor on the expression of carotenoid synthesis genes and the sexM gene

**Fig. 4:**
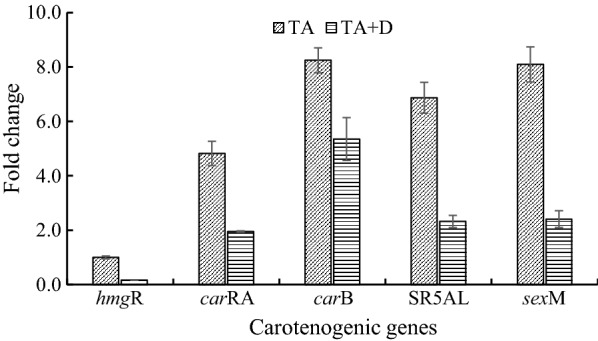
Effects of dutasteride on the expression of carotenogenic genes and the *sex*M gene. TA: Only trisporic acid was added; TA + D: Both trisporic acids and dutasteride were added. *hmg*R gene transcripts with TA treatment was used as the calibrator (value = 1) for relative quantification

Dutasteride belongs to a class of medications called 5α-reductase inhibitors, which can effectively inhibit both the type 1 and type 2 isoforms of steroid 5α-reductase. To determine the roles of the SR5AL gene in lycopene biosynthesis, dutasteride was added at 48 h of fermentation (final concentration: 20 ng/mL). In the presence of trisporic acids, lycopene production in the treatment group with dutasteride was significantly lower than that of the control group. However, the growth of *B. trispora* (−) was not affected because their biomass was not significantly different. To explore the effect of dutasteride at the transcriptional level, the expression of three carotenogenic genes (*hmg*R, *car*RA, and *car*B) and the SR5AL gene were measured by RT-qPCR. As shown in Fig. 4, the fold changes of carotenogenic genes were significantly decreased in the presence of dutasteride. Unexpectedly, the addition of dutasteride also resulted in a significant decrease in the fold change of the SR5AL gene. Dutasteride is a selective and competitive inhibitor of 5-alpha reductase has been widely reported, but its roles in gene expression are unclear.

### Effects of overexpression of the SR5AL gene on the expression of carotenoid synthesis genes and the sexM gene

**Fig. 5:**
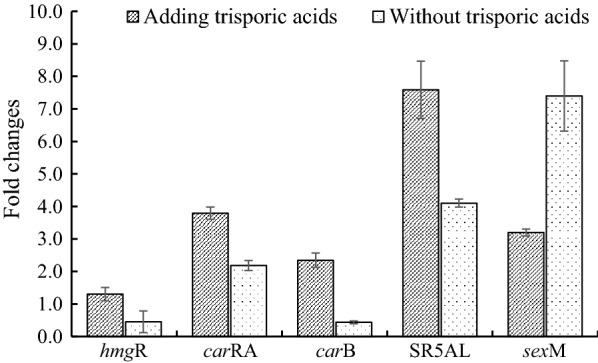
Expression of carotenoid synthesis genes and the *sex*M gene in SR5AL gene-overexpressing transformants **A** with and **B** without trisporic acids. Fold change indicates the ratio of gene expression in the transformant btpHY to that in the transformant btpH. Corresponding genes transcripts in the transformant btpH were used as the calibrator (value = 1) for relative quantification

To reveal the effects of overexpression of the SR5AL gene on lycopene biosynthesis, the cDNA of the SR5AL gene was ligated to an expression plasmid and then transferred into *B. trispora* (−). Transformant *B. trispora* (−) btpHY carrying the expression plasmid containing the SR5AL gene and btpH carrying the empty plasmid were obtained, and their carotenogenic genes and SR5AL gene were measured by RT-qPCR. Compared with transformant btpH, the expression levels of the *car*RA, *car*B, and SR5AL genes were increased 3.95-, 2.27- and 7.86-fold in transformant btpHY in the presence of trisporic acids, respectively (Fig. 5). Without trisporic acids, the fold changes were significantly decreased, although the expression of genes in transformant btpHY was enhanced compared with that in transformant btpH (Fig. 5). The addition of trisporic acids and overexpression of the SR5AL gene had no significant effects on the *hmg*R gene.

## Discussion

Trisporic acids, as sexual recognition and communication signals, cannot be formed in a single culture of the mating type (−) [7]. The absence of trisporic acids is considered the main reason that lycopene cannot be largely formed in nonmated fermentation [5, 14]. This study observed that lycopene production in single fermentation with the addition of a certain amount of trisporic acids by the mating type (−) is almost equal to that in mated fermentation. *B. trispora* (−) is the main lycopene producer, and the role of mating type (+) in lycopene synthesis can be replaced by trisporic acids. In addition, the growth and lycopene biosynthesis of *B. trispora* (−) may be advantageous in single fermentation due to the absence of *B. trispora* (+) to compete for nutrients.

In our previous report, a lycopene-overproducing mutant (WY-239) was obtained by atmospheric and room temperature plasma mutation, and its transcriptome changes from the parent strain were revealed by RNA-seq [18]. Results showed that the carbon metabolism flowed more intensively to acetyl-CoA and then to lycopene in the WY-239 mutant compared with the parent strain. To compare transcriptome changes caused by mutation with that by trisporic acid, the DEGs obtained in this study with those in our previous report were analyzed together. Results showed that very different transcriptional patterns were observed (Additional file 1: Fig. S4). Only a small number of genes were up- or downregulated simultaneously in the two studies, and most of the DEGs were observed in only one study. Some metabolic pathways contained a large number of DEGs in response to the stimulation of trisporic acids, but very few when compared mutant with the parent strain, such as valine, leucine and isoleucine degradation, phosphonate and phosphinate metabolism, and steroid biosynthesis. Sun et al. [14] revealed intracellular biochemical changes in *B. trispora* in response to trisporic acids based on gas chromatography-mass spectrometry. The results showed that the regulation of trisporic acids involved multiple metabolic pathways, such as fatty acids, carbohydrates, and amino acids. Similarly, many primary metabolic pathways were sensitive to the addition of trisporic acids at the transcriptional level observed in this study (Additional file 1: Fig. S5). For example, 13 up-regulated DEGs and 3 down-regulated DEGs were found in the pathways relevant to valine, leucine and isoleucine degradation. The enhancement of this pathway promotes the synthesis of acetyl-CoA and HMG-CoA, which are important precursors for lycopene synthesis. Similar phenomena were found in fatty acid degradation and butanoate metabolism. In secondary metabolic pathways, terpenoid backbone and carotenoid biosynthesis were strengthened, while metabolic branches derived from terpenoid backbone biosynthesis pathways are generally weakened, such as steroid biosynthesis, sesquiterpenoids and triterpenoid biosynthesis, and ubiquinone and other terpenoid-quinone biosynthesis pathways. For example, steroid biosynthesis pathway contains 1 up- and 7 down-regulated DEGs. Since steroids and lycopene share the same precursor–farnesyl pyrophosphate, weakening of steroids biosynthesis helps more farnesyl pyrophosphate to lycopene biosynthesis. From the gene expression changes of these metabolic pathways, trisporic acids facilitated the metabolic flux towards carotenoid biosynthesis.

Of the top 10 up- and downregulated DEGs, Unigene1762_All (encoding carotene oxygenase) and CL1473.Contig2_All (encoding carotenoid cleavage dioxygenase 1) are involved in carotenoid cleavage and trisporic acids biosynthesis [17, 23]. Unigene6321_All (sex determining gene) controls mating and other sexual development [11]. In addition to these DEGs that have been reported to be associated with carotenoid biosynthesis or sexual reproduction, Unigene4532_All (encoding steroid 5α-reductase like gene) is also noteworthy. Steroid-5α-reductase, also known as 3-oxo-5α-steroid 4-dehydrogenases, is an enzyme involved in androgen biosynthesis in mammals (named the SRD5A gene) and brassinosteroid biosynthesis in plants (named the DET2 gene). 5α-Reductase from microbial sources has been reported in only a small number of fungi, such as *Penicillium decumbens*, *P. chrysogenum*, and *Ustilago maydis* (reviewed by Kristan and Rizner) [24]. The function of this enzyme and the metabolic pathways in which it is involved have not been fully elucidated in microorganisms. 5α-Reductase from *Penicillium* spp. can reduce double bonds in testosterone to form 5-DHT and is also inhibited by finasteride and PM-9, competitive inhibitors of the human enzyme [25, 26]. Dutasteride, a 5α-reductase inhibitor used in mammals, clearly inhibited lycopene formation and had no effect on the growth of *B. trispora* (Additional file 1: Fig. S6). We surmise that dutasteride inhibits the activity of the enzyme encoded by the SR5AL gene, making it almost impossible to synthesize lycopene, regardless of the presence of trisporic acids. On the other hand, the overexpression of the SR5AL gene in transformant btpHY significantly increased the expression of lycopene biosynthesis genes. The color of the mycelium of btpHY (carrying the expression plasmid) on solid medium was more intense than that of btpH (carrying the empty plasmid), which indicated that lycopene formation was enhanced because of the overexpression of the SR5AL gene. Therefore, the SR5AL gene is one of the key genes in *B. trispora* (−) responding to trisporic acids based on the following three phenomena: (1) the expression of the SR5AL gene was significantly increased in the presence of trisporic acids, as revealed by both RNA-seq and qPCR; (2) lycopene biosynthesis was blocked when a 5α-reductase inhibitor was added; and (3) lycopene biosynthesis was enhanced due to the overexpression of the SR5AL gene.

The pathway of carotenoid biosynthesis in *B. trispora* is clear, and the SR5AL gene was not observed to be involved. The stimulation of lycopene formation by trisporic acids was sharply reduced in the presence of dutasteride. In addition, the overexpression of the SR5AL gene can at least partially replace the role of trisporic acids. These facts indicated that trisporic acids may not directly act on carotenoid biosynthesis genes, and there are some intermediate substances at work. Sex determination in *B. trispora* is controlled by a small, specialized region of the genome, named *sex*P and *sex*M in the mating type (+) and (−), respectively. The *sex*M gene is also a notable gene in response to trisporic acids. Its expression was significantly increased in the presence of trisporic acids, but the increment was lower under the condition of adding both trisporic acids and dutasteride than when only trisporic acids were added. This phenomenon is consistent with the SR5AL gene. In addition, the overexpression of the SR5AL gene resulted in the upregulation of the *sex*M gene in transformant btpHY, regardless of whether trisporic acids were added. The *sex*P and *sex*M proteins of Mucorales are sex-specific transcription factors, since they contain a sequence similar to the high mobility group domain found in genes responsible for sex determination in many organisms [13, 27]. The expression of the *sex*M gene in *Mucor mucedo* also increased in the presence of trisporic acids or in mated culture [12]. Sexual development results in changes in the transcriptional level of several hundred genes [28].

## Conclusions

In summary, changes in transcriptional levels in *B. trispora* upon exposure to trisporic acids were demonstrated by RNA-seq, and some key regulatory genes were found. Trisporic acids facilitated the metabolic flux towards carotenoid biosynthesis, but their roles may be indirect. The SR5AL gene is one of the key genes for regulating lycopene biosynthesis in *B. trispora* (−) in response to trisporic acids.

## Supplementary Information

**Additional file1**: **Fig. S1** Map of plasmid pBARGPE1-Hygro-SR5AL. **Fig. S2** Comparison of fold changes derived from RNA-seq with that with RT-qPCR for selective genes. **Fig. S3** Top 10 of up- and downregulated DEGs. **Fig. S4** Compare of the numbers of DEGs obtained in this study with that in our previous report. TA-UP, TA-DOWN: up, down-regulated DEGs with trisporic acids treatment (this study). MS-UP, MS-DOWN: up, down-regulated DEGs in mutant strain (previous study). **Fig. S5** Transcriptional changes from glucose to carotenoids. Red upward arrows indicate that upregulated DEGs prevail in the pathway, and blue downward arrows indicate that downregulated DEGs prevail in the pathway. **Fig. S6** Color of mycelia of* B. trispora* (−). a: No exogenous substance was added. b: Both trisporic acids and dutasteride were added. c: Only trisporic acids were added. **Fig. S7** Melt curve of qPCR. A: *car*RA, B: *car*B, C: SR5AL, D: *sex*M. **Fig. S8** Agarose gel electrophoresis of PCR-amplified gene fragments coding for SR5AL gene. Lane 1 and lane 2: wild type; lane 3-8: hygromycin-resistant transformants.

## Data Availability

Raw RNA-seq datasets of *B. trispora* are deposited in the sequence read archive (SRA) database (accession number PRJNA728833). Other datasets used and/or analyzed during the current study are available from the corresponding authors upon reasonable request.

## Abbreviations

SR5AL: Steroid 5α-reductase-like gene
SRA: Sequence read archive
FCs: Fold changes
FDR: False discovery rate
DEGs: Differentially expressed genes
FPKM: Fragments per kilobase per million
GO: Gene ontology
KEGG: Kyoto encyclopedia of genes and genomes
DAVID: Database for annotation, visualization and integrated discovery
