# Supplementary material for: SR5AL serves as a key regulatory gene in lycopene biosynthesis by Blakeslea trispora
Source: Microb Cell Fact. 2022 Jun 25;21:126. doi: 10.1186/s12934-022-01853-x (PMC9233402; doi:10.1186/s12934-022-01853-x)
Supplement: Supplementary file 1 — Additional file1: Fig. S1 Map of plasmid pBARGPE1-Hygro-SR5AL. Fig. S2 Comparison of fold changes derived from RNA-seq with that with RT-qPCR for selective genes. Fig. S3 Top 10 of up- and downregulated DEGs. Fig. S4 Compare of the numbers of DEGs obtained in this study with that in our previous report. TA-UP, TA-DOWN: up, down-regulated DEGs with trisporic acids treatment (this study). MS-UP, MS-DOWN: up, down-regulated DEGs in mutant strain (previous study). Fig. S5 Transcriptional changes from glucose to carotenoids. Red upward arrows indicate that upregulated DEGs prevail in the pathway, and blue downward arrows indicate that downregulated DEGs prevail in the pathway. Fig. S6 Color of mycelia of B. trispora (−). a: No exogenous substance was added. b: Both trisporic acids and dutasteride were added. c: Only trisporic acids were added. Fig. S7 Melt curve of qPCR. A: carRA, B: carB, C: SR5AL, D: sexM. Fig. S8 Agarose gel electrophoresis of PCR-amplified gene fragments coding for SR5AL gene. Lane 1 and lane 2: wild type; lane 3-8: hygromycin-resistant transformants. [file 12934_2022_1853_MOESM1_ESM.docx]

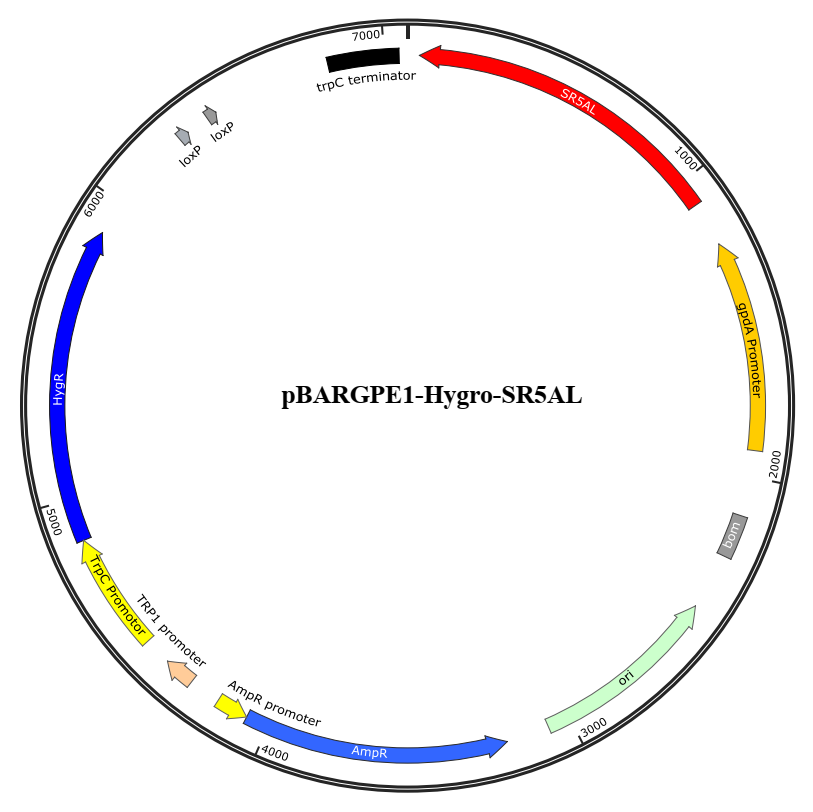


Fig. S1 Map of plasmid pBARGPE1-Hygro-SR5AL


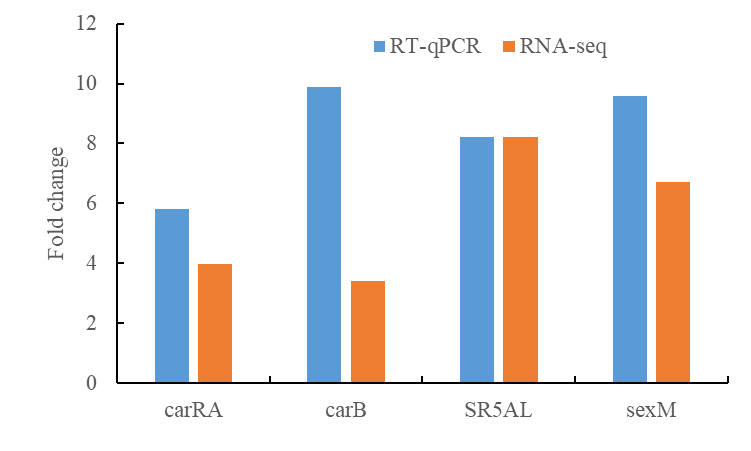


Fig. S2 Comparison of fold changes derived from RNA-seq with that with RT-qPCR for selective genes.


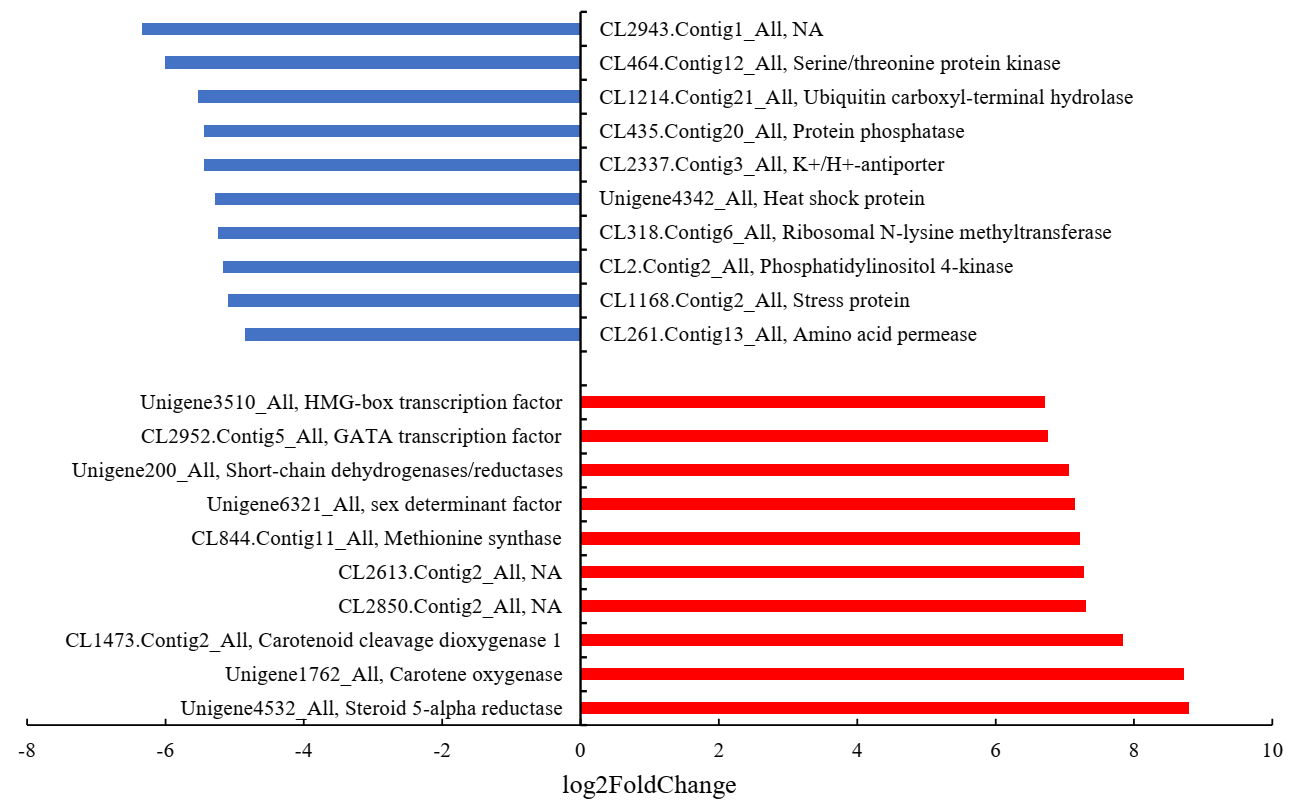


Fig. S3 Top 10 of up- and downregulated DEGs.


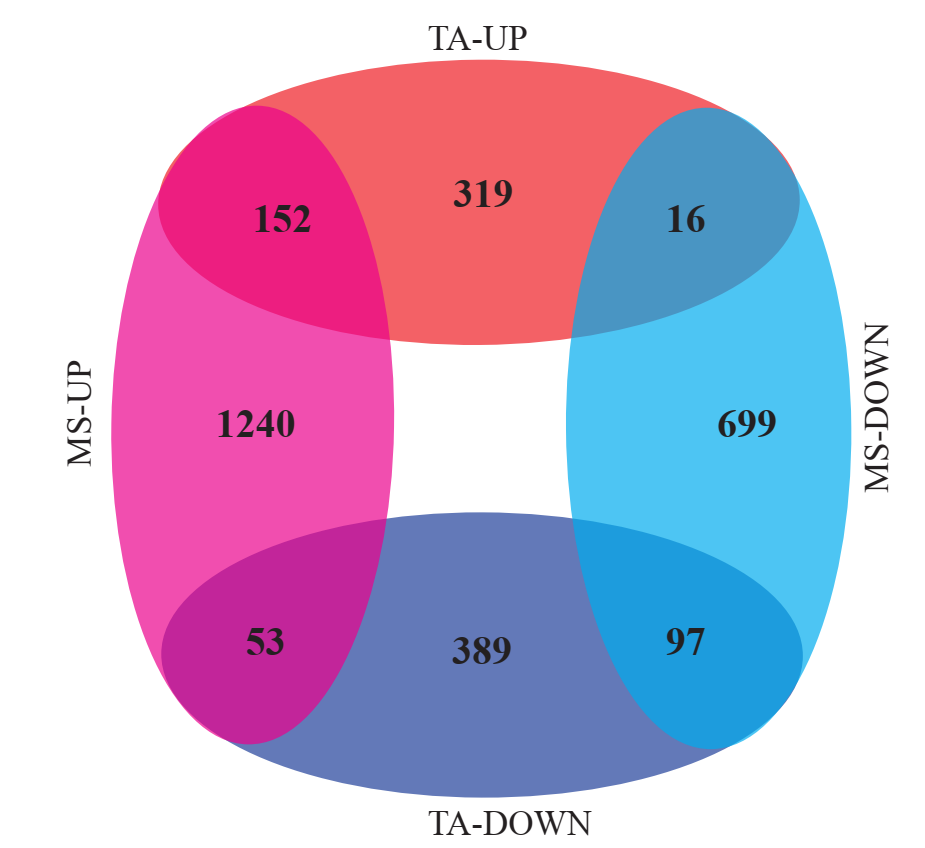


Fig. S4 Compare of the numbers of DEGs obtained in this study with that in our previous report. TA-UP, TA-DOWN: up, down-regulated DEGs with trisporic acids treatment (this study). MS-UP, MS-DOWN: up, down-regulated DEGs in mutant strain (previous study).

Fig. S5 Transcriptional changes from glucose to carotenoids. Red upward arrows indicate that upregulated DEGs prevail in the pathway, and blue downward arrows indicate that downregulated DEGs prevail in the pathway


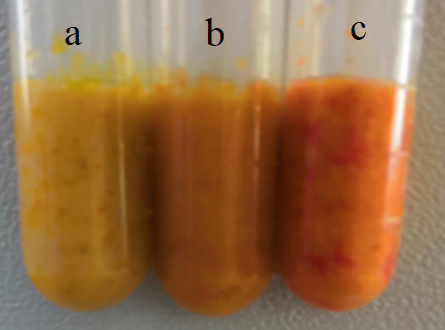


Fig. S6 Color of mycelia of *B. trispora* (-). a: No exogenous substance was added. b: Both trisporic acids and dutasteride were added. c: Only trisporic acids were added.


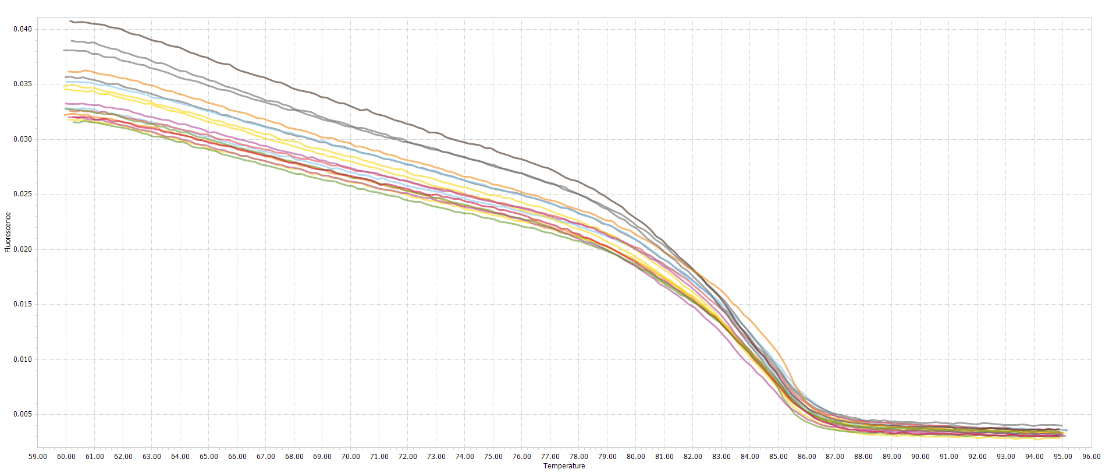


**A**


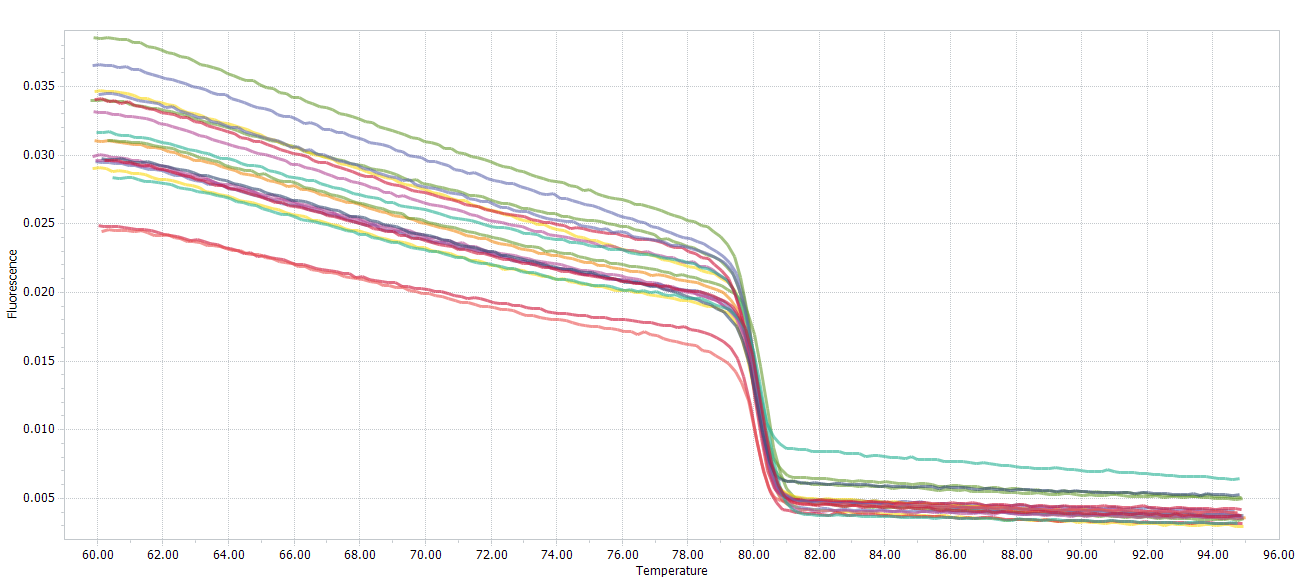


**B**


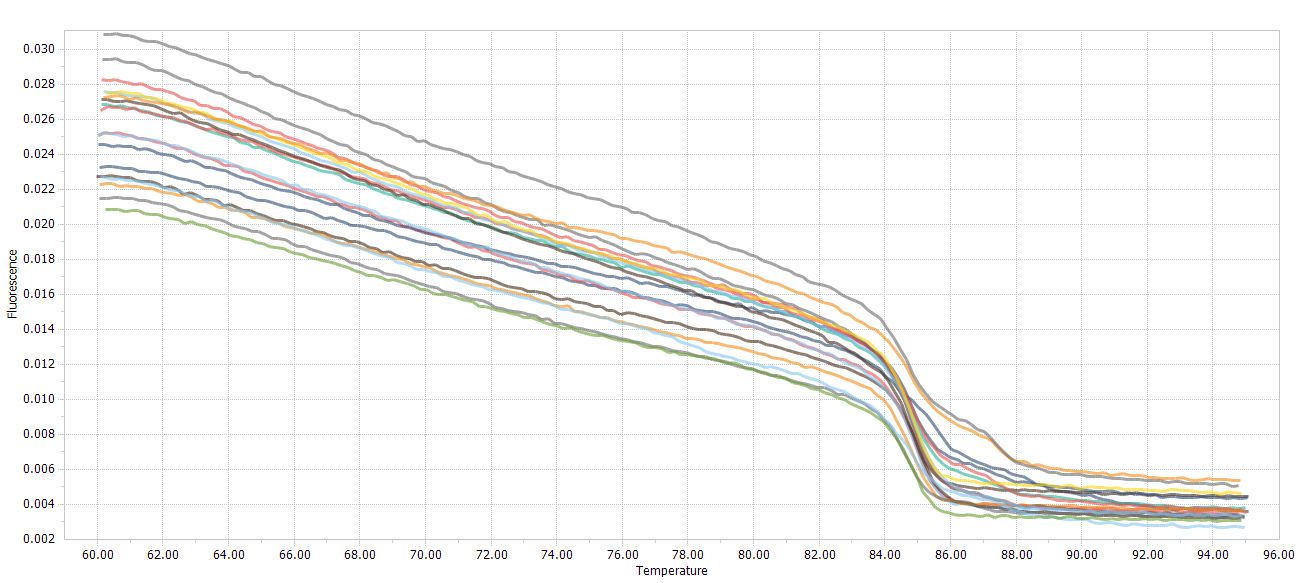


**C**


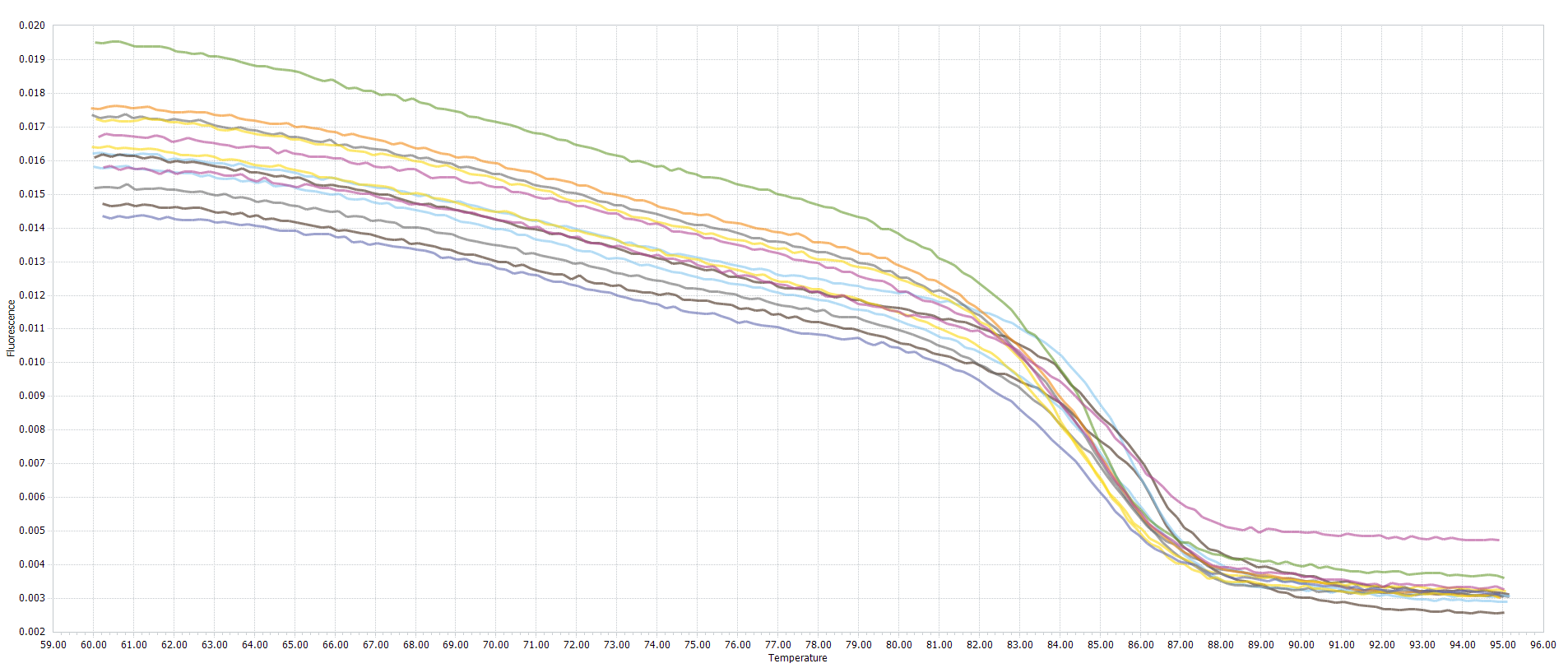


**D**

Fig. S7 Melt curve of qPCR. A: *car*RA, B: *car*B, C: SR5AL, D: *sex*M


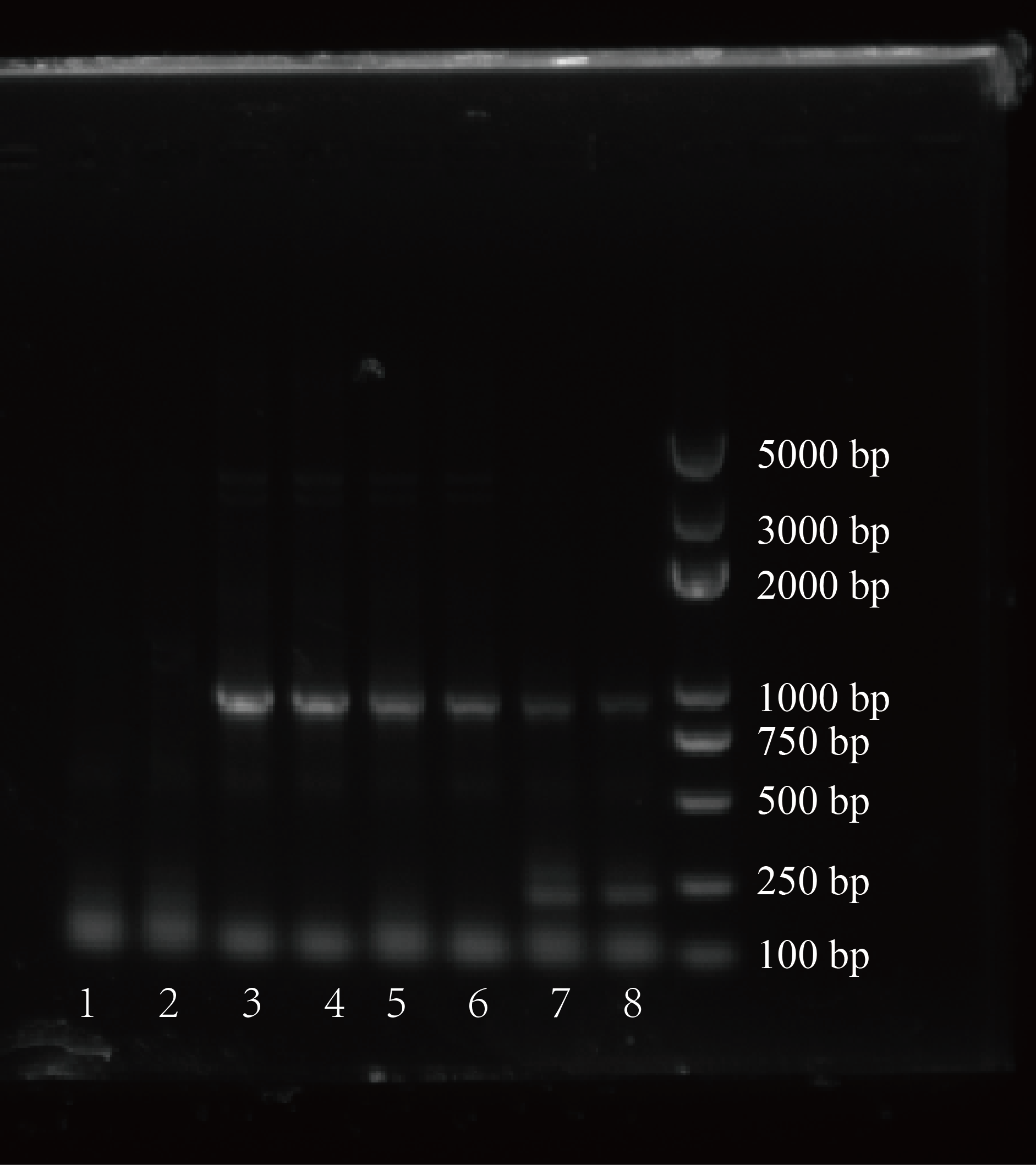


Fig. S8 Agarose gel electrophoresis of PCR-amplified gene fragments coding for SR5AL gene. Lane 1 and lane 2: wild type; lane 3-8: hygromycin-resistant transformants.
